# The association of socio-demographic and environmental factors on childhood diarrhea in Cambodia

**DOI:** 10.12688/f1000research.23246.2

**Published:** 2020-07-03

**Authors:** Vong Pisey, Pannee Banchonhattakit

**Affiliations:** 1 Doctor of Public Health Program, Faculty of Public Health, Khon Kaen University, Khon Kaen, 40002, Thailand; 2 Office of Rural Health Care, Pursat Provincial Department of Rural Development, Ministry of Rural Development, Cambodia; 3 Faculty of Public Health, Khon Kaen University, Khon Kaen, 40002, Thailand

**Keywords:** Socio-demographic, environmental, childhood diarrhea, generalized linear mixed model, Cambodia

## Abstract

**Background:** Diarrhea is still the leading cause of childhood death worldwide, as well as a major cause for concern in developing countries. This study was conducted to investigate the factors related to childhood diarrhea in Cambodia.

**Methods: **A cross-sectional study of the secondary data from the Cambodia Demographic and Health Survey 2014 was conducted using the combination of household data and children’s data. A generalized linear mixed model was used to analyze the determinant factors of childhood diarrhea.

**Results: **The surveys included 2,828 children, aged 12 to 35 months. The prevalence of diarrhea in the last 2 weeks was 16.44% (95% CI: 14.72%-18.31%). Factors with statistically significant associations with childhood diarrhea in Cambodia were: maternal  unemployment, compared with being in employment (AOR = 1.43; 95% CI: 1.14-1.78); the child being male (AOR = 1.25; 95%CI: 1.02-1.53); the presence of unimproved toilet facilities (AOR = 1.17; 95%CI: 1.05-1.31) compared with improved toilet facilities; and unhygienic disposal of children’s stools (AOR = 1.32; 95%CI: 1.06-1.64) compared with hygienic disposal of children’s stools when controlling for other covariates. Both maternal age (one year older; AOR = 0.85; 95%CI: 0.78– 0.93) and child age (one month older; AOR = 0.86; 95%CI: 0.78-0.94) had significant negative associations with the occurrence of childhood diarrhea.

**Conclusion: **Childhood diarrhea remains a public health concern in Cambodia. The probability of diarrhea occurring is shown to be increased by maternal unemployment, the sex of the child being male, lack of provision of improved toilet facilities, and the unhygienic disposal of children’s stools; whereas increasing maternal age and child’s age were associated with a reduced chance of diarrhea occurring. On the basis of these results, we recommend provision of programs focusing on reducing diarrhea through the construction of improved toilet facilities and the promotion of behavior to improve hygiene, specifically targeting younger mothers.

## Introduction

Diarrhea is defined as the passage of loose or watery stools, three or more times each day, or more frequent passage than is normal for an individual ^1^. Diarrhea remains a leading cause of child mortality and morbidity in the world, with an estimated 1.7 billion cases of childhood diarrhea and 525,000 deaths of children under five caused by diarrhea each year ^1, 2^. Diarrhea is the second leading cause of death in children under the age of five years ^1, 2^. Globally, 88% of diarrhea cases are attributable to poor water, poor sanitation or poor hygiene ^3^. There is not just the one single factor associated with childhood diarrhea but multiple factors, including unimproved drinking water sources ^4– 7^, untreated water ^8– 10^, unimproved toilet facilities ^6, 8, 9, 11^, unhygienic disposal of children’s stools ^12– 14^, lack of hand washing facilities ^15, 16^, type and location of residence ^11, 16^, the child’s age ^4, 13, 16^, the child’s sex (male) ^13^, maternal illiteracy ^12, 13, 17^, the mother’s occupation ^9, 12^, maternal age ^14, 18^, wealth index ^4, 19^, and whether or not the child is breastfed ^10, 15^.

In 2014, Cambodia still had one of the highest prevalence levels of diarrhea among children under the age of five amongst countries in South-East Asia, at 12.8% ^20^. By comparison, Myanmar had a prevalence of 10.4% in 2015–16 ^21^, Malaysia 4.4% in 2016 ^7^, Laos 6.5% in 2017 ^22^, Philippines 6.1% in 2017 ^23^, and Indonesia 14.1% in 2017 ^24^. According to 2016 data from UNICEF, Cambodia had 5,947 total neonatal deaths, of which 20 were due to diarrhea; 5,248 post-neonatal deaths, of which 672 were due to diarrhea (13%); and 692 deaths of children under five due to diarrhea (6%) ^25^. High rates of diarrhea alone account for one fifth of the deaths of children under the age of five in Cambodia, and an estimated 10,000 deaths overall each year ^26^. This demonstrates that diarrhea is the most common cause of death in Cambodian children. According to the Cambodia Demographic and Health Survey (CDHS) 2014, the prevalence of diarrhea among children aged 12 to 35 months was high, which is known to affect for child development and growth ^20^.

It is of great importance to understand the factors related to the prevalence of diarrhea among children aged 12 to 35 months. There are no existing studies on the association in this age group, and no national studies on the associated factors with childhood diarrhea in Cambodia have yet been published.

## Methods

### Ethical statement

This research project received approval from the Khon Kean University Ethics Committee in Human Research (HE632097). This study uses existing CDHS data and re-analysis was done under the original consent provided by the participants.

### CDHS 2014

The CDHS 2014 collected data nationally across the country, which is subdivided into 19 province domains. Its sampling frame consisted of 28,455 eligible enumeration areas (EAs), which comprised the 2008 Cambodian General Population Census (GPC). The sample was allocated into urban and rural in each domain with a power allocation preventing oversample urban, and can represent Cambodia is mainly rural. The stratified sample was selected in two stages. In the first stage, a fixed number of EAs were chosen using probabilities weighted proportional to the size of the EA. In the second stage, 24 and 28 households were picked up from every urban cluster and rural cluster, respectively, through a systematic sampling process with equal probability weighting. 15,825 households, 17,578 women, and 5,190 men were interviewed between the 2 ^nd^ June and the 12 ^th^ December 2014; further details can be found in the CDHS 2014 report ^20^. The final sample size comprised 2,828 children aged 12 to 35 months, providing a suitable degree of power (0.9627, 0.9682).

### Data use

Two raw CDHS 2014 datasets, comprising household data and children’s data were combined for use in this study. All entries and variables in these datasets were included in this study.

### Dependent variable

The prevalence of diarrhea is the dependent variable considered in this study. This is referred to the questionnaire thus: “Has (NAME) had diarrhea in the last 2 weeks?” The dichotomous variable *childhood diarrhea* can take values “1” representing a response of “yes” or “0” representing “no” and “don’t know” responses.

### Independent variables

Socio-demographic characteristics take the form of continuous variables such as maternal age, child’s age, and number of household members and categorical variables such as maternal education (no education/primary/secondary/higher), maternal occupation (employed/unemployed), mother’s knowledge of oral rehydration salts (ORS) (good/poor) ^27^, exposure to media (yes/no) ^28^, sex of the child, breastfeeding (ever/never), deworming (yes/no) ^27^, vaccination (ever/never), residence (urban/rural) and wealth index (poorest/poorer/middle/richer/richest) ^27^. CDHS data were organized in 19 province domains, which we regrouped into four regions: Central Plain; Tonle Sap; Coastal and Sea; and Plateau and Mountains ^29^. Environmental characteristics were also treated as categorical variables, including drinking water source (improved/unimproved) ^30^, whether or not the same source of drinking water was used during wet and dry seasons (same/different), whether or not water was treated before drinking (always/no), type of toilet facility (improved/unimproved) ^30^, hygiene (adequate/inadequate) ^30^, and disposal of children’s stools (sanitary/unsanitary) ^31^. World Health Organization (WHO) guidelines on water, sanitation and hygiene (WASH) were used to classify WASH as either improved or unimproved, and sanitary or unsanitary according to the WHO/UNICEF Joint Monitoring Programme ( Table 1 and Table 2) ^30, 31^

**Table 1.** Joint Monitoring Programme classification of improved and unimproved water, sanitation and hygiene (WASH) ^30^.

Service	Improved	Unimproved
Drinking water	Piped water, boreholes or tube wells, protected dug wells, protected springs, rainwater, and packaged or delivered water, and provided collection time is not more than 30 minutes for a round trip, including queuing	Unprotected dug well, unprotected spring, surface water (river, reservoirs, lakes, ponds, streams, canals, and irrigation channels).
Sanitation	Flush and pour flush connected to piped sewer, septic tanks or pit latrines; ventilated improved pit (VIP) latrine, composting toilets or pit latrines with slabs, and that are not shared with other households	Flush and pour flush not to sewer/septic tank/pit latrine, pit latrine without slab/open pit, bucket, hanging toilet/hanging latrine, no facility/bush/field
Hygiene	Availability of a handwashing facility on premises with soap and water	No handwashing facility on premises

**Table 2.** Joint Monitoring Programme classification of sanitary and unsanitary disposal of children stool ^31^.

Sanitary	Unsanitary
Child used toilet or latrine Put or rinsed in the toilet or latrine Buried	Put or rinsed into drain or ditch Throw into the garbage Left in the open or not disposed of Other

### Statistical analysis

Statistical data analyses were performed using STATA/SE 14.0 ^32^ as follows.

Categorical variables were analyzed to provide frequency and percentage. Continuous variables were calculated as means, standard deviations, and ranges. A weighting variable was used in the form of the woman’s individual sample weighting. Cross-tabulations were run with the appropriate sample weights to provide nationally representative results ^19^. The *svyset* command was used to test for complex survey sampling methods used in the original surveys, in order to adjust for differences in the probabilities of sample selection and to avoid using over-sampled strata within the survey data ^27^.

The prevalence of diarrhea was estimated as a percentage. The numerator was the number of living children aged 12 to 35 months with an occurrence of diarrhea during the two weeks preceding the interview (i.e. an answer “yes” to, “Has (NAME) had diarrhea in the last 2 weeks?”) and the denominator was the number of living children aged 12 to 35 months.

A bivariate analysis with simple logistic regression was performed using the *svyset* ( *svy* command). A linearity test was conducted between the continuous variable and dependent variable. Any independent variables significant at p<0.25 were entered into the initial model ^33, 34^. Multicollinearity assessment was performed with the independent variables-variance inflation factor (VIF) ^35^. Finally, a multivariate analysis was performed using a generalized mixed linear model with four regions picked as ‘random effects’ corresponding to the various clusters in the sampling design ^36^. The backward stepwise procedure was applied as the model fitting strategy. Statistical significance was considered at a threshold of p<0.05 and the adjusted odds ratio (AOR) with 95% confidence intervals (CI) was considered as the magnitude of the effect.

## Results

A total of 2,828 children were included in the study. The majority of the children (84.12%) lived in rural areas. Nearly half (44.03%) lived in Central Plain and one third (33.32%) lived in Tonle Sap. The mean of maternal age was 28.27±5.89 years old. More than half the mothers (51.08%) attended primary school. Three quarters (75.10%) of the mothers were employed and the average number of household members was five. More than half (51.18%) of the children were male and the mean age was 23.33±6.79 months. Almost all (96.17%) children had been breastfed; 59.60% had received deworming treatment. Out of 2,828 households, more than half (54.07%) always had treated water to drink; 57.97% had an unimproved toilet facility; while 68.01% used adequate hygiene; and 70.25% used sanitary disposal of children’s stool ( Table 3).

**Table 3.** Socio-demographic and environmental characteristics of households in Cambodia, 2014 (n=2,828).

Variables	Frequency	Percentage
***Maternal characteristics***		
**Age (years)**		
16–24	397	14.04
25–34	1591	56.26
35–49	840	29.70
Mean±SD	28.27±5.89	
Range	16 to 49	
**Education**		
No education	366	12.96
Primary	1445	51.08
Secondary	921	32.58
Higher	96	3.38
**Occupation**		
Employed	2124	75.10
Unemployed	704	24.90
**Knowledge of oral rehydration** **salts**		
Good	2717	96.05
Poor	111	3.95
**Exposure to media**		
Yes	1808	63.92
No	1020	36.08
***Children’s characteristics***		
**Age (months)**		
12–23	1460	51.64
24–35	1368	48.36
Mean±SD	23.33±6.79	
Range	12 to 35	
**Sex**		
Male	1448	51.18
Female	1381	48.82
**Breastfeeding status**		
Ever	2720	96.17
Never	108	3.83
**Deworming**		
Yes	1686	59.60
No	1142	40.40
***Household characteristics***		
**Residence**		
Urban	449	15.88
Rural	2379	84.12
**Region**		
Coastal and Sea	169	5.98
Tonle Sap	942	33.32
Central Plain	1245	44.03
Plateau and Mountains	472	16.67
**Number of household members**		
1–4	969	34.28
>4	1859	65.72
Mean±SD	5.73±2.31	
Range	1 to 22	
**Wealth index**		
Poorest	672	23.76
Poorer	523	18.49
Middle	550	19.44
Richer	493	17.45
Richest	590	20.86
***Environmental characteristics***		
**Drinking water during dry** **season**		
Improved	1745	61.71
Unimproved	1083	38.29
**Drinking water during wet** **season**		
Improved	2320	82.02
Unimproved	508	17.98
**Same source of drinking water** **during wet and dry season**		
Same	1955	69.11
Different	873	30.89
**Treating water to drink**		
Yes, always	1529	54.07
No	1299	45.93
**Toilet facility**		
Improved	1189	42.03
Unimproved	1640	57.97
**Hygiene**		
Adequate	1923	68.01
Inadequate	905	31.99
**Disposal of children’s stool**		
Sanitary	1987	70.25
Unsanitary	841	29.75

SD, standard deviation.

### Bivariate analysis of factors associated with childhood diarrhea in Cambodia

Factors with a significant association with childhood diarrhea (p<0.05) were maternal age, maternal occupation, the child’s age, available toilet facilities, and the method of stool disposal ( Table 4). Further, the factors of the child’s sex, the number of household members, wealth index, source of drinking water during dry season, whether or not the same source of drinking water is used during wet and dry seasons, and the treatment/non-treatment of drinking water did not reach significance but did meet the pre-determined threshold of p<0.25 for inclusion in the initial model. Finally, region (p<0.25) also met the criteria for inclusion in the initial model and was used as a random effect. As such, the multivariate analysis was conducted using a generalized mixed linear model with each of the four regions of Cambodia treated as random effects.

**Table 4.** Bivariate analysis of factors associated with childhood diarrhea in Cambodia, 2014 (n=2,828).

Variables	Number	Diarrhea %	COR	95% CI	p-value
**Overall**	2828	16.44		14.72-18.31	
**Maternal age (years)**	2828	N/A	0.82	0.73-0.92	<0.001
**Maternal education**					0.681
Literate	2462	16.29	1		
Illiterate	366	17.46	1.09	0.73-1.62	
**Maternal occupation**					0.007
Employed	2124	15.00	1		
Unemployed	704	20.78	1.49	1.11-1.98	
**Mother’s knowledge of oral** **rehydration salts**					0.481
Good	2717	16.61	1		
Poor	111	12.21	0.69	0.25-1.90	
**Mother’s exposure to media**					0.502
Yes	1808	15.99	1		
No	1020	17.23	1.09	0.84-1.42	
**Child’s age (months)**	2828	N/A	0.83	0.75-0.92	<0.001
**Child’s sex**					0.075
Female	1381	14.86	1		
Male	1448	17.94	1.25	0.97-1.61	
**Breastfeeding status**					0.268
Ever	2720	16.64	1		
Never	108	11.42	0.64	0.29-1.40	
**Deworming**					0.504
Yes	1686	16.91	1		
No	1142	15.75	0.91	0.71-1.17	
**Residence**					0.561
Urban	449	15.39	1		
Rural	2379	16.64	1.10	0.80-1.50	
**Region**					0.203
Coastal and Sea	169	12.36	1		
Tonle Sap	942	15.55	1.31	0.82-2.07	
Central Plain	1245	16.92	1.44	0.92-2.25	
Plateau and Mountains	472	18.40	1.60	1.02-2.51	
**Number of household** **members**					0.095
>4	1859	15.38	1		
1–4	969	18.47	1.25	0.96- 1.62	
**Wealth index**					0.128
Richest	590	14.44	1		
Richer	493	17.40	1.25	0.82-1.90	
Middle	550	14.65	1.02	0.67-1.55	
Poorer	523	14.50	1.00	0.67-1.50	
Poorest	672	20.46	1.52	1.03-2.26	
**Drinking water during dry** **season**					0.065
Improved	1745	15.12	1		
Unimproved	1083	18.56	1.28	0.98-1.66	
**Drinking water during wet** **season**					0.676
Improved	2320	16.27	1		
Unimproved	508	17.22	1.07	0.78-1.48	
**Same source of drinking water** **during wet and dry season**					0.161
Same	1955	15.56	1		
Different	873	18.40	1.22	0.92-1.62	
**Treating water to drink**					0.139
Yes, always	1529	15.28	1		
No	1299	17.81	1.20	0.94-1.53	
**Toilet facility**					0.013
Improved	1189	13.61	1		
Unimproved	1640	18.49	1.20	1.04-1.39	
**Hygiene**					0.995
Adequate	1923	16.44	1		
Inadequate	905	16.43	0.99	0.74-1.34	
**Disposal of children’s stool**					0.020
Sanitary	1987	14.99	1		
Unsanitary	841	19.85	1.40	1.05-1.87	

COR, crude odds ratio; CI, confidence interval.

### Multivariate analysis of factors associated with childhood diarrhea in Cambodia

The multivariate analysis ( Table 5) showed that as maternal age increased by a year, the odds of the child suffering from diarrhea decreased 15% (AOR = 0.85; 95%CI: 0.78– 0.93; p=0.001). The odds of suffering from diarrhea was 43% higher (AOR = 1.43; 95% CI: 1.14-1.78; p=0.002) in children whose mother was unemployed compared to employed. As the child’s age increased by a month, the odds of the child suffering from diarrhea decreased 14% (AOR = 0.86; 95%CI: 0.78-0.94; p=0.001). The odds of suffering from diarrhea was 25% higher (AOR = 1.25; 95%CI: 1.02-1.53; p=0.031) in males compared to females. The odds of suffering from diarrhea was 17% higher (AOR = 1.17; 95%CI: 1.05-1.31; p=0.004) in children living in a household with unimproved toilet facilities compared with those with improved toilet facilities. The odds of suffering from diarrhea was 32% higher (AOR = 1.32; 95%CI: 1.06-1.64; p=0.011) in children whose stools were disposed of unhygienically compared to children whose stools were disposed of hygienically.

**Table 5.** Multivariate analysis of factors associated with childhood diarrhea in Cambodia, 2014 using generalized mixed linear model (n=2,828).

Variables	Number	Diarrhea %	AOR	95% CI	p-value
**Maternal age (years)**	2828	N/A	0.85	0.78-0.93	0.001
**Maternal occupation**					0.002
Employed	2124	15.00	1		
Unemployed	704	20.78	1.43	1.14-1.78	
**Child’s age (months)**	2828	N/A	0.86	0.78-0.94	0.001
**Child’s sex**					0.031
Female	1381	14.86	1		
Male	1448	17.94	1.25	1.02-1.53	
**Toilet facility**					0.004
Improved	1189	13.61	1		
Unimproved	1640	18.49	1.17	1.05-1.31	
**Disposal of children’s stool**					0.011
Sanitary	1987	14.99	1		
Unsanitary	841	19.85	1.32	1.06-1.64	

AOR, adjusted odds ratio; CI, confidence interval.

## Discussion

This is the first study to report factors associated with diarrhea in children aged 12 to 35 months at the national level in Cambodia. Younger maternal age, maternal unemployment, younger child age, being male, lack of unimprovement to toilet facilities, and unhygienic disposal of children’s stools were found to be associated with childhood diarrhea.

Socio-demographic characteristics such as maternal age were significantly associated with reduced incidence of diarrhea, in line with studies conducted in Brazil and Tanzania ^14, 18^, and perhaps due to the mother having more experience in childcare and feeding. The association of maternal unemployment with the incidence of diarrhea is consistent with a study in Senegal ^9^. The child’s age had a significant, negative association with incidence of diarrhea, in line with many studies in Ethiopia and Tanzania ^4, 14, 16^, and potentially due to the development of the immune system throughout childhood. Males were more likely to suffer from diarrhea than females, which may simply reflect a natural predisposition of males to develop diarrhea more frequently than females ^37^, but is also supported by a previous study conducted in India ^13^.

Environmental characteristics such as the lack of improvements to toilet facilities were significantly associated with the incidence of diarrhea, consistent with many studies including a systematic review ^4, 6, 8, 11^. Finally, disposal of children’s stools was significantly associated with the incidence of diarrhea, consistent with previous studies in Ethiopia, India, and Tanzania ^12– 14^. These findings demonstrate that the quality of sanitation facilities strongly influences the prevalence of childhood diarrhea in Cambodia.

A limitation of this research study was that it used a cross-sectional design with just one outcome measure (diarrhea prevalence) taken as a snapshot at a given point in time. Future longitudinal studies may improve on this. The CDHS 2014 was not fully comprehensive in that it did not cover the WASH factors of hand washing before preparing meals and after defecating. The inclusion of these questions in the survey would give a more comprehensive analysis of hygiene practices in the population. Moreover, self-reporting measures and recall bias could happened and considered in the study. Further, the CDHS 2014 captured data by household, rather than by individual person, which may introduce a confound in that it has a tendency to under-estimate the quality of both drinking water source and sanitation facility available.

## Conclusion

Diarrhea still remains a public health concern among children in Cambodia. The probability of developing diarrhea is strongly associated with maternal unemployment, being male, not having access to improved toilet facilities, or practicing hygienic disposal of children’s stools. Conversely, increasing maternal and child age is associated with a reduction in the probability of developing diarrhea.

## Recommendations

”Based on this finding, the authors provide the following recommendations.

*National*: The WASH program should focus on younger mothers, mothers of younger children and unemployed mothers. Guidance should include sanitary methods of stool disposal, water treatment, sanitation, and health. Intervention programs should focus on the construction of improved toilet facilities and promoting hygienic behaviors.

*Local*: Younger mothers should be encouraged to enroll in health education. Additional community sanitation facilities should be constructed and existing facilities should be improved/maintained.

*Future study*: Longitudinal studies are needed to measure the impact of these interventions.

## Data availability

Our study used raw children’s and household data from DHS, Cambodia 2014. Data are free to access for research purposes and can be obtained through the DHS Program after registering and obtaining an approval letter from the Inner City Fund (ICF) ( https://dhsprogram.com/data/Access-Instructions.cfm).
