# Supplementary material for: The association of socio-demographic and environmental factors on childhood diarrhea in Cambodia
Source: F1000Res. 2021 Sep 23;9:303. Originally published 2020 Apr 28. [Version 5] doi: 10.12688/f1000research.23246.5 (PMC8499042; doi:10.12688/f1000research.23246.5)
Supplement: Peer review history

# Peer review: Reviewer response for version 5

**DOI:** 10.5256/f1000research.77795.r95139

**Published:** 2021-10-07

**Authors:** Siyan Yi

**Affiliations:** 1 Saw Swee Hock School of Public Health, National University of Singapore, Singapore, Singapore; 2 KHANA Center for Population Health Research, Phnom Penh, Cambodia; 3 Center for Global Health Research, Touro University California, Vallejo, CA, USA

The authors have addressed all the critical points. I have no further comments.

Is the work clearly and accurately presented and does it cite the current literature?

Partly

If applicable, is the statistical analysis and its interpretation appropriate?

Yes

Are all the source data underlying the results available to ensure full reproducibility?

Yes

Is the study design appropriate and is the work technically sound?

Yes

Are the conclusions drawn adequately supported by the results?

Yes

Are sufficient details of methods and analysis provided to allow replication by others?

Partly

Reviewer Expertise:

Epidemiology, community-based intervention and evaluation, infectious diseases

I confirm that I have read this submission and believe that I have an appropriate level of expertise to confirm that it is of an acceptable scientific standard.

# Peer review: Reviewer response for version 5

**DOI:** 10.5256/f1000research.77795.r95138

**Published:** 2021-10-07

**Authors:** Okechukwu S. Chukwudeh

**Affiliations:** 1 North-West University, Mahikeng, South Africa

The article is a topical issue especially in less developed countries. It is presumed it will make meaningful impact to scholars and policy makers.

Is the work clearly and accurately presented and does it cite the current literature?

Partly

If applicable, is the statistical analysis and its interpretation appropriate?

Partly

Are all the source data underlying the results available to ensure full reproducibility?

No

Is the study design appropriate and is the work technically sound?

Partly

Are the conclusions drawn adequately supported by the results?

No

Are sufficient details of methods and analysis provided to allow replication by others?

No

Reviewer Expertise:

Sociology (Demography and Population Studies)

I confirm that I have read this submission and believe that I have an appropriate level of expertise to confirm that it is of an acceptable scientific standard.

# Peer review: Reviewer response for version 4

**DOI:** 10.5256/f1000research.59156.r91452

**Published:** 2021-08-26

**Authors:** Okechukwu S. Chukwudeh

**Affiliations:** 1 North-West University, Mahikeng, South Africa

**Report on the manuscript titled “The association of socio-demographic and environmental factors on childhood diarrhea in Cambodia”**

**Abstract**

 The abstract should be filtered to avoid repetition of points

**Methods**

1. **Study design and setting**: The design, settings, location and relevant dates was not clearly stated. This should be stated in the methodology section. Where is Central Plain, and Tonle Sap? These were not described in the methodology. Are they cities or communities in rural or urban clusters?
2. **Participants**: Eligibility criteria was totally missing in this study. Kindly fulfil this requirement. The Author should provide the population size. What is the total number of under-five children (0-5 years) in the country? What is the total number of children 12 to 35 months in the country? What is significant about 12 to 35 months in Cambodia?
3. **Variables**: The variables, indicators, predictors and confounders for the study are not clearly outline. Kindly outline this in alignment with recent WASH analyses pattern e.g Water ladder scale, and Sanitation ladder services (this should be added to what author have on the work).
4. **Data**: The data for each variable of measurement is entirely missing. Kindly provide sources of data for each variables and indicators.
5. **Bias**: Kindly provide efforts to address potential bias
6. **Study size**: kindly explain how the sample size was arrived at. How did you arrive at the sample of 2,828? You selected 2,828 sample which represent 565 households based on five persons per household. Recall that you stated that the total number of households was 15,825. This means you selected 4% of the entire household in the study location. This is small and this affects the generalization of the study. Kindly review this along side the eligibility criteria. Also, how were 15,825 households, 17,578 women, and 5,190 men interviewed? What is the significant of interviewing men? The study sample size is clumsy. Kindly review.
7. **Statistical method**: How were variables handled in the analyses. The statistical method was not well explained. What is the meaning of improve and unimproved sanitation? Describe the statistical method and sampling strategy used for the study. How were data handled? What kind of technique was used to calculate the prevalence of diarrhea (see abstract)?

**Conclusion**: The method for the study is not sufficiently detailed to understand the approach. The author is advised to provide detailed approach and statistical techniques applied.

**Results**

1. The demographics of the respondents was not sufficiently reported.
2. Descriptive statistics was used for the study. Recall in the methodology, under the 2 ^nd^ stage of the sampling technique, 24 and 28 households were picked from urban and rural clusters, respectively using equal probability weighting. However, result shows that 84% of the children live in rural areas. Kindly align this narrative.
3. Kindly explain the result succinctly.

**Conclusion**: The result should be aligned with the methodology and conclusion

**Interpretations/Discussion**

1. Contextually, why are male more susceptible to diarrhea when other studies such as Nwokocha, Chukwudeh, and Damian (2020) have found otherwise. Kindly justify this contextually, this makes the work unique.
2. If male gender is uniquely susceptible to diarrhea as stated in this study, why was it not included in the conclusion and its implications reflected in the recommendation. However, gender status and disease prevalence are contextual and highly contested in literature.
3. Validity of the results was not included.
4. The results were not engaged with other studies to know the gap that was filled in this study.
5. The contribution to knowledge of the study is not clearly outline in the body of the work.
6. The implication and contribution of findings to the body of knowledge is missing.

**Conclusion**: Author should derive conclusion from the results

**Recommendation**: Invitation to revise (more work is required to satisfy all criteria)

Is the work clearly and accurately presented and does it cite the current literature?

Partly

If applicable, is the statistical analysis and its interpretation appropriate?

Partly

Are all the source data underlying the results available to ensure full reproducibility?

No

Is the study design appropriate and is the work technically sound?

Partly

Are the conclusions drawn adequately supported by the results?

No

Are sufficient details of methods and analysis provided to allow replication by others?

No

Reviewer Expertise:

Sociology (Demography and Population Studies)

I confirm that I have read this submission and believe that I have an appropriate level of expertise to confirm that it is of an acceptable scientific standard, however I have significant reservations, as outlined above.

## References

[1] : Prevalence and the social contexts of childhood diarrhea in Ibadan slums, Southwest Nigeria. *African Journal of Gender, Society and Development (formerly Journal of Gender, Information and Development in Africa)* .2020;9(3) : 10.31920/2634-3622/2020/9n3a1 11-34 10.31920/2634-3622/2020/9n3a1

# Response

**Published:** 2021-09-18

**Authors:** PISEY VONG

**Affiliations:** Ministry of Rural Development, Cambodia, Cambodia

Thank you for your valuable comments to improve the quality of our paper.

#Response to reviewer: Okechukwu S. Chukwudeh

**Abstract**

 The abstract should be filtered to avoid repetition of points

Reply: Taken care.

**Methods**

**1. Study design and setting:** The design, settings, location and relevant dates was not clearly stated. This should be stated in the methodology section. Where is Central Plain, and Tonle Sap? These were not described in the methodology. Are they cities or communities in rural or urban clusters?

Reply: Taken care. Please, see Figure 1 as well as please, kindly see the CDHS 2014 and Data use.

**2. Participants:** Eligibility criteria was totally missing in this study. Kindly fulfil this requirement. The Author should provide the population size. What is the total number of under-five children (0-5 years) in the country? What is the total number of children 12 to 35 months in the country? What is significant about 12 to 35 months in Cambodia?

Reply: Taken care.

**3. Variables:** The variables, indicators, predictors and confounders for the study are not

 clearly outline. Kindly outline this in alignment with recent WASH analyses pattern e.g Water ladder scale, and Sanitation ladder services (this should be added to what author have on the work).

Reply: The authors have retained our originals. Please feel free to check it in limitation.

**4. Data:** The data for each variable of measurement is entirely missing. Kindly provide sources of data for each variables and indicators.

Reply: Some of the variable is not provided the sources because it is the secondary data which has been grouped or categorized already. i.e. source of drinking water during wet and dry seasons (same/different), residence (urban/rural), etc.

**5. Bias:** Kindly provide efforts to address potential bias

Reply: The authors have retained our originals. Please feel free to check it in limitation.

**6. Study size:** kindly explain how the sample size was arrived at. How did you arrive at the sample of 2,828? You selected 2,828 sample which represent 565 households based on five persons per household. Recall that you stated that the total number of households was 15,825. This means you selected 4% of the entire household in the study location. This is small and this affects the generalization of the study. Kindly review this along side the eligibility criteria. Also, how were 15,825 households, 17,578 women, and 5,190 men interviewed? What is the significant of interviewing men? The study sample size is clumsy. Kindly review.

Reply: DHS used large survey which is not only interviewing for only one outcome. It provides information related to fertility, family planning, maternal and child health, gender, HIV/AIDS, malaria, and nutrition. Therefore, you will get the answer. Please, kindly understand the DHS/CDHS report.

**7. Statistical method**: How were variables handled in the analyses. The statistical method was not well explained. What is the meaning of improve and unimproved sanitation? Describe the statistical method and sampling strategy used for the study. How were data handled? What kind of technique was used to calculate the prevalence of diarrhea (see abstract)?

Reply: Our statistical method is already explained. The meaning of improved and unimproved sanitation was outlined in Table 1 and Table 2. Please, kindly see. Our outcome calculation was also outline there. Please, see it carefully.

**Conclusion:** The method for the study is not sufficiently detailed to understand the approach. The author is advised to provide detailed approach and statistical techniques applied.

Reply: As we explain above in statistical method, the authors have retained our originals in this point.

**Interpretations/Discussion**

 1. Contextually, why are male more susceptible to diarrhea when other studies such as Nwokocha, Chukwudeh, and Damian (2020) have found otherwise. Kindly justify this contextually, this makes the work unique.

Reply: The authors confirm that our analysis is correct. We also outline in Discussion that “…. may simply reflect a natural predisposition of males to develop diarrhea more frequently than females ^37^)” as well as a study published in PMC Public Health by Bawankule, R., Singh, A., Kumar, K. *et al*. found the same as our study.

 2. If male gender is uniquely susceptible to diarrhea as stated in this study, why was it not included in the conclusion and its implications reflected in the recommendation. However, gender status and disease prevalence are contextual and highly contested in literature.

Reply: Taken care.

 3. Validity of the results was not included.

Reply: Please, feel free to check it carefully.

 4. The results were not engaged with other studies to know the gap that was filled in this study.

Reply: Please, feel free to check it carefully.

 5. The contribution to knowledge of the study is not clearly outline in the body of the work.

Reply: Please, feel free to check it carefully.

 6. The implication and contribution of findings to the body of knowledge is missing.

Reply: Please, feel free to check it carefully.

**Conclusion:** Author should derive conclusion from the results.

Reply: Please, feel free to check it carefully.

**Recommendation:** Invitation to revise (more work is required to satisfy all criteria)

Reply: Taken care.

# Peer review: Reviewer response for version 4

**DOI:** 10.5256/f1000research.59156.r91191

**Published:** 2021-08-13

**Authors:** Siyan Yi

**Affiliations:** 1 Saw Swee Hock School of Public Health, National University of Singapore, Singapore, Singapore; 2 KHANA Center for Population Health Research, Phnom Penh, Cambodia; 3 Center for Global Health Research, Touro University California, Vallejo, CA, USA

**General comments:**

 This is an interesting study of an important health topic in low-income countries using large datasets from the Cambodia Demographic and Health Survey (CDHS) 2014. These data are often under use in many resource-limited countries, despite their large scope and scale with standardized methods.  Despite its significant contribution to the literature, the manuscript requires substantial improvement in the writing quality and careful proofreading to eliminate several grammatical errors, misuse of punctuations, and complex sentences easily found throughout the text for improving its accuracy and readability. Below are some suggestions that are not exhaustive.

**Abstract**

1. When talking about health-related issues in children, please be specific about age groups. The term ‘childhood diarrhea’ is too broad, while diarrheal diseases commonly affect younger children.
2. The first sentence in background is not very accurate and can be simplified as follows: “Diarrheal diseases remain the leading cause of death among under-five children in lower- and lower-middle-income countries.”
3. Methods: “We analyzed cross-sectional data from the Cambodia Demographic and Health Survey 2014 using combined household and children’s datasets.”
4. Results: The 1 ^st^ sentence: “The survey included 2,828 children aged 12 to 35 months.” and the 3 ^rd^ sentence: “Factors significantly associated with childhood diarrhea were…” - Please correct the misuse and inconsistent use of punctuation.
5. Conclusions: The 2 ^nd^ sentence only repeats the results. Please summarize the key findings without redundancies. Recommendations can be simplified, avoiding unnecessary wording: “Intervention programs should focus on reducing diarrheal diseases by constructing improved toilet facilities and promoting behavior to improve hygiene, specifically targeting younger mothers.”
6. Keywords can also be improved.

**Introduction**

 The writing quality of the introduction needs substantial improvement. I am trying to help, but it is difficult and time-consuming. Here are some examples:

1. The 1 ^st^ sentence: the definition of diarrhea is not very important in the background as everyone is familiar with the basic definitions, and it takes unnecessary space. However, the authors should provide its operational definition specific to this study in methods. This would ensure the reader that the measurement of the outcome variable was valid.
2. The 2 ^nd^ and 3 ^rd^ sentences, paragraph 1, tell almost the same thing and should be combined.
3. The 5 ^th^ sentence, paragraph 1: consider, “Childhood diarrhea is associated with multiple factors, including…”
4. The writing quality makes the background very confusing. For example, the authors stated that, in 2016, 20/5,947 (0.34%) total neonatal deaths were due to diarrhea; 672/5,248 post-neonatal deaths were due to diarrhea (13%); and 692 deaths of children under five due to diarrhea (6%). These are hard to understand – how were ‘neonatal deaths, post-neonatal deaths, and under-five child deaths’ defined? Was it possible that the age groups can be overlapping? The authors continued by stating that “High rates of diarrhea alone account for one fifth of the deaths of children under the age of five in Cambodia, and an estimated 10,000 deaths overall each year.” One-fifth means 20%, which is much higher than any percentages mentioned above.
5. The rationale of study is also relatively weak. For example, the authors stated that, “It is of great importance to understand the factors related to the prevalence of diarrhea among children aged 12 to 35 months.” Please elaborate why it is important to study diarrhea among children in this age group. This should also be explained in the methods (study population).
6. The study’s objective is missing. Please provide a clear objective at the end of the introduction.

**Methods**

1. Sampling procedures: it is great that the authors described the CDHS-2014’s sampling methods. However, the reader needs the information on how the research team reached children aged 12 to 35 months, this study’s population.
2. Please provide operational definition of diarrhea used in the CDHS 2014. This would ensure the reader that the measurement of the outcome variable was valid.
3. I understand that the sample size was sufficiently large to address the research question. However, it would make more sense to mention minimum required sample size calculation, rather than saying that “The final sample size comprised 2,828 children aged 12 to 35 months, providing a suitable degree of power (0.9627, 0.9682),” which was not understandable.
4. Statistical analyses: The sentence, “Any independent variables significant at p<0.25 were entered into the initial model” is inaccurate and incomplete. Consider: “Variables associated with diarrhea in bivariate analyses at a level of p<0.25 were included in the initial model.”
  Also, what did ‘the initial model mean?’
  Later, the authors mentioned, “Finally, a multivariable analysis was performed using…” This confused the reader – was the earlier-mentioned model different from the multivariable analysis (model)?

**Results**

1. The 1 ^st^ sentence is redundant with that in methods (CDHS 2014 description).
2. The result presentation style (mean ± SD) has not been recommended for use since long ago. Please use ‘mean (SD xx)’ format.
3. Much of the bivariate results presentation only repeated the data analysis methods. The authors better keep the space to clarify the direction of the bivariate associations.
4. The way p-values are presented in tables does not follow a standardized format in scientific/academic papers.

**Discussion**

1. The discussion section is superficial, mostly only compared this study’s findings to those from the literature without in-depth discussions of the key findings and their policy implication. 
2. Conclusions and recommendations should be combined. The recommendations can be summarized to the extent to which this study’s findings can support.

Is the work clearly and accurately presented and does it cite the current literature?

Partly

If applicable, is the statistical analysis and its interpretation appropriate?

Yes

Are all the source data underlying the results available to ensure full reproducibility?

Yes

Is the study design appropriate and is the work technically sound?

Yes

Are the conclusions drawn adequately supported by the results?

Yes

Are sufficient details of methods and analysis provided to allow replication by others?

Partly

Reviewer Expertise:

Epidemiology, community-based intervention and evaluation, infectious diseases

I confirm that I have read this submission and believe that I have an appropriate level of expertise to confirm that it is of an acceptable scientific standard, however I have significant reservations, as outlined above.

# Response

**Published:** 2021-09-18

**Authors:** PISEY VONG

**Affiliations:** Ministry of Rural Development, Cambodia, Cambodia

Thank you so much for your valuable comments to improve the quality of our paper.

#Response to reviewer: Siyan Yi

**Abstract**

 1. When talking about health-related issues in children, please be specific about age groups.

 The term ‘childhood diarrhea’ is too broad, while diarrheal diseases commonly affect younger children.

Reply: Taken care

 2. The first sentence in background is not very accurate and can be simplified as follows:

 “Diarrheal diseases remain the leading cause of death among under-five children in lower and lower-middle-income countries.”

Reply: Taken care

 3. Methods: “We analyzed cross-sectional data from the Cambodia Demographic and Health Survey 2014 using combined household and children’s datasets.”

Reply: Taken care

 4. Results: The 1st sentence: “The survey included 2,828 children aged 12 to 35 months.” and the 3rd sentence: “Factors significantly associated with childhood diarrhea were…” - Please correct the misuse and inconsistent use of punctuation.

Reply: Taken care

 5. Conclusions: The 2 ^nd^ sentence only repeats the results. Please summarize the key findings without redundancies. Recommendations can be simplified, avoiding unnecessary wording: “Intervention programs should focus on reducing diarrheal diseases by constructing improved toilet facilities and promoting behavior to improve hygiene, specifically targeting younger mothers.”

Reply: Taken care

 6. Keywords can also be improved.

Reply: Taken care: Prevalence, Child, Diarrhea, Cross-Sectional Study, Cambodia

 Introduction

 The writing quality of the introduction needs substantial improvement. I am trying to help, but it is difficult and time-consuming. Here are some examples:

 1. The 1 ^st^ sentence: the definition of diarrhea is not very important in the background as everyone is familiar with the basic definitions, and it takes unnecessary space. However, the authors should provide its operational definition specific to this study in methods. This would ensure the reader that the measurement of the outcome variable was valid.

Reply: Taken care.

 2. The 2 ^nd^ and 3 ^rd^ sentences, paragraph 1, tell almost the same thing and should be combined.

A: Taken care

 3. The 5 ^th^ sentence, paragraph 1: consider, “Childhood diarrhea is associated with multiple factors, including…”

Reply: Taken care

 4. The writing quality makes the background very confusing. For example, the authors stated that, in 2016, 20/5,947 (0.34%) total neonatal deaths were due to diarrhea; 672/5,248 postneonatal deaths were due to diarrhea (13%); and 692 deaths of children under five due to diarrhea (6%). These are hard to understand – how were ‘neonatal deaths, post-neonatal deaths, and under-five child deaths’ defined? Was it possible that the age groups can be overlapping? The authors continued by stating that “High rates of diarrhea alone account for one fifth of the deaths of children under the age of five in Cambodia, and an estimated 10,000 deaths overall each year.” One-fifth means 20%, which is much higher than any percentages mentioned above.

Reply: Taken care

4.1. Neonatal death: Probability of dying during the first 28 days of life

Post-neonatal deaths: Probability of dying between 28 and 364 days of age

Under-five death: Probability of dying between birth and exactly 5 years of age

***Sources:*** *https://data.unicef.org/topic/child-survival/neonatal-mortality/* and

*https://www-doh.state.nj.us/doh-shad/view/sharedstatic/PostneonatalMortalityRate.pdf*

4.2. The authors can confirm that the age groups cannot be overlapping.

4.3. “ *High rates of diarrhea alone account for one fifth of the deaths of children under the age of five in Cambodia, and an estimated 10,000 deaths overall each year*.” is the report from UNICEF year 2014; however, “ *in 2016, 20/5,947 (0.34%) total neonatal deaths….” *is the report from UNICEF year 2018. Therefore, the authors would like to reverse the sentence as following:

“According to report from UNICEF Cambodia year 2014, high rates of diarrhea alone account for one fifth of the deaths of children under the age of five in Cambodia, and an estimated 10,000 deaths overall each year. However, according to report from UNICEF year 2018, by year 2016 Cambodia had 5,947 total neonatal deaths, of which 20 were due to diarrhea; 5,248 post-neonatal deaths, of which 672 were due to diarrhea (13%); and 692 deaths of children under five due to diarrhea (6%).”

 5. The rationale of study is also relatively weak. For example, the authors stated that, “It is of great importance to understand the factors related to the prevalence of diarrhea among children aged 12 to 35 months.” Please elaborate why it is important to study diarrhea among children in this age group. This should also be explained in the methods (study population).

Reply: Taken care

 6. The study’s objective is missing. Please provide a clear objective at the end of the introduction.

Reply: Taken care

**Methods**

 1. Sampling procedures: it is great that the authors described the CDHS-2014’s sampling methods. However, the reader needs the information on how the research team reached children aged 12 to 35 months, this study’s population.

Reply: Taken care: In our analysis, we included only children aged 12 to 35 months (n=2,828) due to the high prevalence of diarrhea among this aged group while comparing with other age groups and this age period is known to affect for child development and growth.”

 2. Please provide operational definition of diarrhea used in the CDHS 2014. This would ensure the reader that the measurement of the outcome variable was valid.

Reply: Taken care.

 3. I understand that the sample size was sufficiently large to address the research question. However, it would make more sense to mention minimum required sample size calculation,

 rather than saying that “The final sample size comprised 2,828 children aged 12 to 35

 months, providing a suitable degree of power (0.9627, 0.9682),” which was not

 understandable.

Reply: The authors used secondary data for this study; so, authors should test for the power of its sample size whether it is sufficient for this study analysis. This following paper is also performed https://bmjpaedsopen.bmj.com/content/5/1/e000992 the power to clarify its sufficiency. According to our analysis it is unable to show how minimum the sample size is.

 4. Statistical analyses: The sentence, “Any independent variables significant at p<0.25 were entered into the initial model” is inaccurate and incomplete. Consider: “Variables associated with diarrhea in bivariate analyses at a level of p<0.25 were included in the initial model.”

Reply: Taken care

 Also, what did ‘the initial model mean?’

Reply: Taken care. Multivariable model

 Later, the authors mentioned, “Finally, a multivariable analysis was performed using…” This confused the reader – was the earlier-mentioned model different from the multivariable analysis (model)?

Reply: Taken care. It is the same

**Results**

 1. The 1 ^st^ sentence is redundant with that in methods (CDHS 2014 description).

Reply: Taken care

 2. The result presentation style (mean ± SD) has not been recommended for use since long ago. Please use ‘mean (SD xx)’ format.

Reply: Taken care

 3. Much of the bivariate results presentation only repeated the data analysis methods. The authors better keep the space to clarify the direction of the bivariate associations.

Reply: Taken care

 4. The way p-values are presented in tables does not follow a standardized format in scientific/academic papers.

Reply: We think reviewer suggest to present p-value in each group. We agree with the reviewer if our research question in which the variable has stratified group *i.e. Whether age group of each gender associated on Outcome (Y) or not*. *Then, we must stratify group of each age group and gender; after that we will present each p-value in each group.* In our research question, we better to present p-value of each factor because we would like to know each factor. Therefore, the authors have retained our originals.

**Discussion**

 1. The discussion section is superficial, mostly only compared this study’s findings to those from the literature without in-depth discussions of the key findings and their policy implication.

Reply: Taken care

 2. Conclusions and recommendations should be combined. The recommendations can be summarized to the extent to which this study’s findings can support.

Reply: Taken care

# Peer review: Reviewer response for version 1

**DOI:** 10.5256/f1000research.25665.r64014

**Published:** 2020-06-15

**Authors:** Siyan Yi

**Affiliations:** 1 Saw Swee Hock School of Public Health, National University of Singapore, Singapore, Singapore; 2 KHANA Center for Population Health Research, Phnom Penh, Cambodia; 3 Center for Global Health Research, Touro University California, Vallejo, CA, USA

General comments: This study used data from Cambodia Demographic and Health Surveys to identify risk factors associated with diarrhea in children aged 12 to 35 months. Overall, the study findings are interesting and may contribute to the literature in this area given the scarcity of data in low- and middle-income countries. The analyses appeared appropriate. The quality of the writing is acceptable, although more careful proofreading is required before the paper can be published.

 Here are my specific comments:

**Title:**

1. The term 'influence' may not be appropriate for this study as it can only tell the associations of variables, not causal relationships.

**Abstract:**

1. Methods: The first sentence "A cross-sectional study was conducted using the combination of two datasets from the Cambodia Demographic and Health Survey 2014" is hard to understand. The authors may want to make it clear that this study used secondary data from CDHS. What are the two data sets?
2. What was the time frame for the prevalence of diarrhea - past month or lifetime?

**Introduction:**

1. Please provide a reference for the statement in the second sentence, paragraph 1.
2. Avoid starting sentences with numbers.
3. It would be helpful for readers if authors could define some terminology; e.g., neonatal deaths, post-neonatal deaths, under-five deaths, etc.
4. Paragraph 2:
  - "...and 692 deaths of children under five due to diarrhea (6%)." What is the denominator of the 6%? In the following sentence, the authors stated that 'diarrhea alone account for one fifth of the deaths of children under the age of five in Cambodia." Please clarify these.
  - "High rates of diarrhea alone account for...' Diarrhea alone?
  - It is confusing that this study used data from CDHS 2014, but also cited the prevalence of diarrhea in the same population and from the same data, while claiming that no national studies on childhood diarrhea in Cambodia have yet been published. - The rationale of the study needs improvement.

**Methods:**

1. What 'province domains' mean?
2. What does this mean: '...,which comprised the 2008 Cambodian General Population Census (GPC)?'
3. 'The sample considered any domain...' is not understandable.
4. Although the CDHS 2014 was referred to, some variables require a clear definition; e.g., improved/unimproved water sources, toilet facilities, adequate/inadequate hygiene, sanitary/unsanitary disposal of children's stools, etc.
5. Data analyses:
  - It is not accurate to state this "Continuous data were treated as means, standard deviations, and ranges for analysis." Perhaps something like 'For continuous variables, mean and standard deviations were calculated..."
  - I am not sure what authors wanted to tell by this "A weighting variable was used in the form of the woman’s individual sample weighting."
  - Any independent variables significant at p<0.25 in bivariate analyses were entered into the initial model.
  -Multicolinearity assessment was performed...

**Results:**

1. ...and one third (33.32%) in Tonle Sap region?
2. It should be mean (SD xx).
3. "More than half the mothers (51.08%) attended primary school." Did this include mothers who had no education?
4. Any details to define the breastfeeding - duration, exclusivity...?
5. Please check this data: "...and 77.97% of them had never been vaccinated." This could be very wrong as the immunization coverage in Cambodia has been globally recognized as very high.

**Discussion:**

1. This section can be improved by extending more in-depth literature in this area and link to the policy implication of the findings.
2. Further limitations of the study should also be included (e.g., self-reporting measures, recall bias...).
3. Conclusions and recommendations can be combined.
4. Recommendations can be summarized.

Is the work clearly and accurately presented and does it cite the current literature?

Partly

If applicable, is the statistical analysis and its interpretation appropriate?

Yes

Are all the source data underlying the results available to ensure full reproducibility?

Yes

Is the study design appropriate and is the work technically sound?

Yes

Are the conclusions drawn adequately supported by the results?

Yes

Are sufficient details of methods and analysis provided to allow replication by others?

Partly

Reviewer Expertise:

Epidemiology, community-based intervention and evaluation, infectious diseases

I confirm that I have read this submission and believe that I have an appropriate level of expertise to confirm that it is of an acceptable scientific standard, however I have significant reservations, as outlined above.

# Response

**Published:** 2020-06-29

**Authors:** PISEY VONG

**Affiliations:** Ministry of Rural Development, Cambodia, Cambodia

Review1

**Title:**

 The term 'influence' may not be appropriate for this study as it can only tell the associations of variables, not causal relationships.

 A: Agree with the reviewer. As suggested, we have revised the term to “The association”. See title, para 1 on page 1

**Abstract:**

 1. Methods: The first sentence "A cross-sectional study was conducted using the combination of two datasets from the Cambodia Demographic and Health Survey 2014" is hard to understand. The authors may want to make it clear that this study used secondary data from CDHS. What are the two data sets?

 A: Taken care. "A cross-sectional study of the secondary data from the Cambodia Demographic and Health Survey (CDHS) 2014 was conducted using the combination of household data and children’s data. See Abstract, 1 ^st^ sentence, para 2 on page 1

 2. What was the time frame for the prevalence of diarrhea - past month or lifetime?

 A: Taken care. The authors added the text “in the last 2 weeks”. See Abstract, 2 ^nd^ sentence, para 3 on page 1

**Introduction:**

 1. Please provide a reference for the statement in the second sentence, paragraph

 A: Taken care. “1,2”. See 2 ^nd^ sentence, para 1 on page 3.

 2. Avoid starting sentences with numbers.

  A: Taken care. Globally, 88% of diarrhea cases are attributable…… See 4 ^th^ sentence, para 1 on page 3.

 3. It would be helpful for readers if authors could define some terminology; e.g., neonatal deaths, post-neonatal deaths, under-five deaths, etc.

 A: We are sorry, we do not agree with the peer reviewer on this. Authors have retained our original because we think it is not much helpful to add terminology of neonatal deaths, post-neonatal deaths, under-five deaths.

 4. Paragraph 2:

 - "...and 692 deaths of children under five due to diarrhea (6%)." What is the denominator of the 6%? In the following sentence, the authors stated that 'diarrhea alone account for one fifth of the deaths of children under the age of five in Cambodia." Please clarify these.

 - "High rates of diarrhea alone account for...’Diarrhea alone?

 - It is confusing that this study used data from CDHS 2014, but also cited the prevalence of diarrhea in the same population and from the same data, while claiming that no national studies on childhood diarrhea in Cambodia have yet been published. - The rationale of the study needs improvement.

 A: The denominator of the 6% is the “number of under five children”, however in the data from UNICEF, they do not put it. They just put only “under five deaths due to diarrhea: 692”; and “% underfive deaths due to diarrhoea: (6%)”. According to calculation by the authors, denominator of (6%) is about 11,533.

 A: Please, see the reference on number 26 which mentioned as “High incidences of diarrhoeal diseases alone account for one fifth of the deaths of children age five and under in Cambodia, and an estimated 10,000 overall deaths annually, largely owing to lack of sanitation and poor hygiene practices”.

 A: Agree with the reviewer. As suggested, we have revised the text to “There are no existing studies on the association in this age group, and no national studies on the associated factors with childhood diarrhea in Cambodia have yet been published”. See 2 ^nd^ sentence, para 3 on page 3.

**Methods:**

 1.What 'province domains' mean?

 A. Province domains means the 19 sampling domains of provinces. There are 24 provinces in Cambodia in CDHS 2014, of which fourteen individual provinces (Banteay Meanchey, Kampong Cham, Kampong Chhnang, Kampong Speu, Kampong Thom, Kandal, Kratie, Phnom Penh, Prey Veng, Pursat, Siem Reap, Svay Rieng, Takeo, and Otdar Meanchey); and five groups of provinces (Battambang and Pailin, Kampot and Kep, Preah Sihanouk and Koh Kong, Preah Vihear and Stung Treng, and Mondul Kiri and Ratanak Kiri)

 2. What does this mean: '..., which comprised the 2008 Cambodian General Population Census (GPC)?'

 A. It means “which used the 2008 Cambodian General Population Census”

  That has been mentioned in CDHS 2014 as “The sampling frame used for the 2014 CDHS was derived from the list of all enumeration areas (EAs) created for the 2008 Cambodia General Population Census (GPC), provided by NIS

 3. The sample considered any domain...' is not understandable.

 A: Taken care. We have revised the text to “The sample was allocated into urban and rural in each domain with a power allocation preventing oversample urban, and can represent Cambodia is mainly rural. See 3 ^rd^ sentence, para 3 on page 3.

 4. Although the CDHS 2014 was referred to, some variables require a clear definition; e.g., improved/unimproved water sources, toilet facilities, adequate/inadequate hygiene, sanitary/unsanitary disposal of children's stools, etc.

 A: Agree with the reviewer. As suggested, we have added the text “World Health Organization (WHO) guidelines on water, sanitation and hygiene (WASH) were used to classify WASH as either improved or unimproved according to the WHO/UNICEF Joint Monitoring Programme (Table1 and Table 2). See 3 ^rd^ sentence, para 2 on page 4; and we also added the Table 1 and Table 2. See on page 11 and 12.

 5. Data analyses:

 - It is not accurate to state this "Continuous data were treated as means, standard deviations, and ranges for analysis." Perhaps something like 'For continuous variables, mean and standard deviations were calculated..."

 - I am not sure what authors wanted to tell by this "A weighting variable was used in the form of the woman’s individual sample weighting."

 - Any independent variables significant at p<0.25 in bivariate analyses were entered into the initial model.

 -Multicolinearity assessment was performed...

 A: Agree with the reviewer. As suggested, we have revised the text “Categorical variable were analyzed to provide frequency and percentage. Continuous variable were calculated as means, standard deviations, and ranges”. See 1 ^st^ sentence, para 3 on page 4.

 A: Because it is survey data, by providing national representative, we used Woman’s individual weighting because child data was accessed by asking for their mother.

 A: Yes, please see in the “Result” on 2 ^nd^ sentence, para 2 on page 6.

 A: Taken care “Multicolinearity assessment was performed...”

**Results:**

 ...and one third (33.32%) in Tonle Sap region?

 A. Taken care. We have revised by adding “lived”. See on 2nd sentence, para 1 page 5

 2. It should be mean (SD xx).

 A. Taken care.

 3."More than half the mothers (51.08%) attended primary school." Did this include mothers who had no education?

 A: No, It did not include mothers who had no education

 4. Any details to define the breastfeeding - duration, exclusivity...?

 A: No, in the data showing only “ever breastfed (not currently breastfeed, never breastfed, still breastfeeding”; so we group as “ever and never”.

 5. Please check this data: "...and 77.97% of them had never been vaccinated." This could be very wrong as the immunization coverage in Cambodia has been globally recognized as very high.

 A: Agree with the reviewer. As in the data of 2828 showed that 2.15% was *no*, 22.03% was *yes*, 0.27% was *don’t know*, and 75.55 was *missing*. The authors request to delete this variable since it is too much missing, moreover according to literature review this variable is not related with childhood diarrhea, only rotavirus vaccination is associated.

 Discussion:

 1. This section can be improved by extending more in-depth literature in this area and link to the policy implication of the findings.

 A: Agree with the reviewer. However, we do not extending more in-depth literature. We have retained our original.

 2. Further limitations of the study should also be included (e.g., self-reporting measures, recall bias...).

  A. Taken care. See 4th sentence, para 3 on page 7.

 3. Conclusions and recommendations can be combined.

  A: Authors have retained our original because it will be a good idea to separate the limitation, recommendations and conclusion. Moreover, conclusion is an important part of the paper.

 4. Recommendations can be summarized.

 A: Taken care. As suggested, the authors have summarized the text in Recommendations part. See page 8.
